# Evaluation of Hepatotoxicity with Treatment Doses of Flucytosine and Amphotericin B for Invasive Fungal Infections

**DOI:** 10.1155/2016/5398730

**Published:** 2016-02-02

**Authors:** Alexandra Folk, Coralia Cotoraci, Cornel Balta, Maria Suciu, Hildegard Herman, Oana Maria Boldura, Sorina Dinescu, Lucian Paiusan, Aurel Ardelean, Anca Hermenean

**Affiliations:** ^1^Department of Pathology, Faculty of Medicine, “Vasile Goldis” Western University of Arad, 86 Rebreanu Street, 310414 Arad, Romania; ^2^Department of Bioethics, Faculty of Medicine, “Vasile Goldis” Western University of Arad, 86 Rebreanu Street, 310414 Arad, Romania; ^3^Institute of Life Sciences, Vasile Goldis Western University of Arad, 86 Rebreanu, 310414 Arad, Romania; ^4^Faculty of Veterinary Medicine, Department of Chemistry, Biochemistry and Molecular Biology, Banat University of Agricultural Sciences and Veterinary Medicine “King Mihai I of Romania” Timisoara, 119 Calea Aradului, 300645 Timisoara, Romania; ^5^Department of Biochemistry and Molecular Biology, University of Bucharest, 91-95 Splaiul Independentei, 050095 Bucharest, Romania; ^6^Department of Histology, Faculty of Medicine, “Vasile Goldis” Western University of Arad, 86 Rebreanu Street, 310414 Arad, Romania

## Abstract

Invasive fungal infection is a well-known cause of morbidity and mortality in immunocompromised patients. In this study we aimed to evaluate the hepatotoxicity induced by combined therapy of flucytosine and amphotericin B, at three different doses administered to mice for 14 days: 50 mg/kg flucytosine and 300 *μ*g/kg amphotericin B; 100 mg/kg flucytosine and 600 *μ*g/kg amphotericin B; 150 mg/kg flucytosine and 900 *μ*g/kg amphotericin B. Liver injuries were evaluated by analysis of optic and electron microscopy samples, changes in TNF-*α*, IL-6, and NF-*κ*B inflammation markers levels of expression, and evaluation of mRNA profiles. Histological and ultrastructural analysis revealed an increase in parenchymal and portal inflammation in mice and Kupffer cells activation. Combined antifungal treatment stimulated activation of an inflammatory pathway, demonstrated by a significant dose-dependent increase of TNF-*α* and IL-6 immunoreactivity, together with mRNA upregulation. Also, NF-*κ*B was activated, as suggested by the high levels found in hepatic tissue and upregulation of target genes. Our results suggest that antifungal combined therapy exerts a synergistic inflammatory activation in a dose-dependent manner, through NF-*κ*B pathway, which promotes an inflammatory cascade during inflammation. The use of combined antifungal therapy needs to be dose limiting due to the associated risk of liver injury, especially for those patients with hepatic dysfunction.

## 1. Introduction

Invasive fungal infection is a well-known cause of morbidity and mortality in immunocompromised patients, including those with transplantation, hematological malignancies, or corticosteroid therapy.

Three classes of antifungal agents are extensively used for treatment of systemic fungal infections: polyenes, azoles, and echinocandins. The polyene class of antifungal drugs is divided into conventional polyenes (nystatin, amphotericin B-deoxycholate) and lipid-based formulations of amphotericin B. Polyenes are recommended in cases of intolerance to azoles or echinocandins, when the organism becomes resistant to other agents, or in the case of uncertainty infection due to non-*Candida* yeast [1].

Amphotericin B was the first antifungal drug available to treat systemic fungal infections, commonly used in cases of most yeasts and filamentous fungi, disseminated candidiasis, and invasive pulmonary aspergillosis [2]. Its primary mechanism of action acts via binding to ergosterol, located in the cell membrane of sensitive fungi, followed by increase of cell membrane permeability and possibly leading to cell death [3]. Meanwhile, the efficacy of amphotericin B is filled up by the drug's ability to stimulate macrophages [4].

Flucytosine is a synthetic antimycotic compound without intrinsic antifungal capacity. After intake, it is converted to 5-fluorouracil and then to metabolites that inhibit fungal RNA and DNA synthesis [5]. Flucytosine monotherapy is used only in chromoblastomycosis and vaginal and lower urinary tract candidosis [5]. In all other situations, flucytosine is used together with other agents, for systemic fungal infections caused by sensitive organisms, because of the rapid emergence of resistance when used alone. The combination of flucytosine with amphotericin B has been shown to be beneficial in clinical study with cryptococcal meningitis [6] and its use is recommended [7]. Penetration of the drug into the eye and central nervous system is good [8].

The incidence rates of treatment discontinuation due to adverse reactions associated with antifungal therapy were higher than 10% for amphotericin B and itraconazole and 2.5% to 3.8% for fluconazole, caspofungin, and micafungin, whereas 1.5% of the patients stopped itraconazole treatment due to hepatotoxicity [9]. Furthermore, hepatotoxicity without treatment discontinuation was recorded for voriconazole and flucytosine monotherapy that ranges between 0 and 25% [5, 10].

Based on the well-established side-effect profiles for both monotherapies of flucytosine and amphotericin B, we aimed to evaluate the hepatotoxicity induced by combined therapy, at increasing doses. In the present study, we hypothesized that toxicity of flucytosine and amphotericin B coadministration is dose limiting via cytokine-mediated proinflammatory activity.

## 2. Material and Methods

### 2.1. Materials

Amphotericin B was purchased from Bristol-Myers Squibb (Saint-Remy-sur-Avre, France) and flucytosine (Ancotil, MEDA Pharma, Paris, France). Anti-TNF-*α*, IL-6, and NF-*κ*B, antibodies were supplied from Santa Cruz Biotechnology (Santa Cruz, CA, USA) and Novocastra kit for immunohistochemistry was purchased from Leica Microsystems (Germany).

### 2.2. Animals and Experimental Procedure

Adult male CD1 mice weighing 25 ± 3 g were obtained from Animal Facility of “Vasile Goldis” Western University of Arad, Romania. All experimental procedures were approved by the Ethical Committee of “Vasile Goldis” Western University of Arad.

The mice were divided into four groups:

 Group 1: control mice received by gavage the equivalent volume of 0.9% normal saline solution for 14 days.
 Group 2: mice received 50 mg/kg flucytosine by gavage and were intraperitoneally injected with 300 *μ*g/kg amphotericin B for 14 days.
 Group 3: mice received 100 mg/kg flucytosine by gavage and were intraperitoneally injected with 600 *μ*g/kg amphotericin B for 14 days.
 Group 4: mice received 150 mg/kg flucytosine by gavage and were intraperitoneally injected with 900 *μ*g/kg amphotericin B for 14 days.

The selection of three doses of combined flucytosine-amphotericin B cotreatment was based on previous published reports, related to systemic antifungal efficacy [5, 11, 12].

Mice were sacrificed 24 hours after the last administration and liver samples were used for histopathology, immunohistochemistry, and electron microscopy.

### 2.3. Histopathology

The liver was cut into small pieces and fixed in 4% paraformaldehyde. Following dehydration in an ascending series of ethanol, the tissue samples were cleared in toluene, embedded in paraffin, and sliced into 5 *μ*m sections. The sectioned samples were stained with haematoxylin and eosin (H&E). Sections were examined using an Olympus BX43 microscope and photographed using a digital camera (Olympus XC30).

### 2.4. Immunohistochemistry

Immunohistochemical studies were performed on paraffin embedded liver tissue sections of 5 *μ*m thickness, previously deparaffinized, and rehydrated by using a standard technique. Rabbit polyclonal anti-TNF-*α*, IL-6, and NF-*κ*B p65 diluted 1 : 100 (Santa Cruz, CA, USA) were used as primary antibodies. Immunoreactions were visualized employing Novocastra (Leica Biosystems, Germany), Peroxidase/DAB kit, according to the manufacturer's instructions. Negative control sections were processed by substitution of primary antibodies with irrelevant immunoglobulins of matched isotype, used in the same conditions as primary antibodies. Stained slides were analyzed by light microscopy (Olympus BX43, Hamburg, Germany).

### 2.5. RT-PCR Analysis

Total RNA from liver and kidney mouse tissues was isolated and purified using* SV Total RNA Isolation System* (Promega), following the manufacturer's instructions. Quantity and quality of purified RNA were assessed using a NanoDrop 8000 spectrophotometer (Thermo Scientific). cDNA was synthesized from 2 *μ*g of total RNA using First Strand cDNA Synthesis Kit (Thermo Scientific) and oligo d(T)_8_ primer. 2 *μ*L of RT-PCR reaction results was used as template in qPCR experiments. Real-time PCR was performed using Luminaris HiGreen qPCR Master Mix, low ROX (Thermo Scientific) with Mx3000P real-time PCR system. Samples were run in triplicate. The primers sequences and all experimental validation data were obtained from the PrimerBank website: http://pga.mgh.harvard.edu/primerbank/. Primers were synthesized by Eurogentec, Belgium (Table 1).

The ratio for the gene expression was normalized using GAPDH. Results were interpreted by 2^ΔΔC(T)^ method [13].

### 2.6. Electron Microscopy

EM liver samples were prefixed with 2.7% glutaraldehyde solution in 0.1 M phosphate buffer at 4°C for 1.5 h, washed in 0.15 M phosphate buffer (pH 7.2), and postfixed in 2% osmic acid solution in 0.15 M phosphate buffer. Dehydration was performed in acetone and samples were embedded in the epoxy embedding resin Epon 812. Thick sections of 60 nm were cut with Leica EM UC7 ultramicrotome and analyzed with a TEM Tecnai 12 Biotwin electron microscope.

### 2.7. Statistical Analysis

Statistical analysis was conducted with a one-way ANOVA using Stata 13 software (StataCorp LP, Texas, USA). A value of *P* < 0.05 was considered to be statistically significant.

## 3. Results

### 3.1. Flucytosine and Amphotericin B Coadministration Induced Histopathological Changes

**Figure 1:**
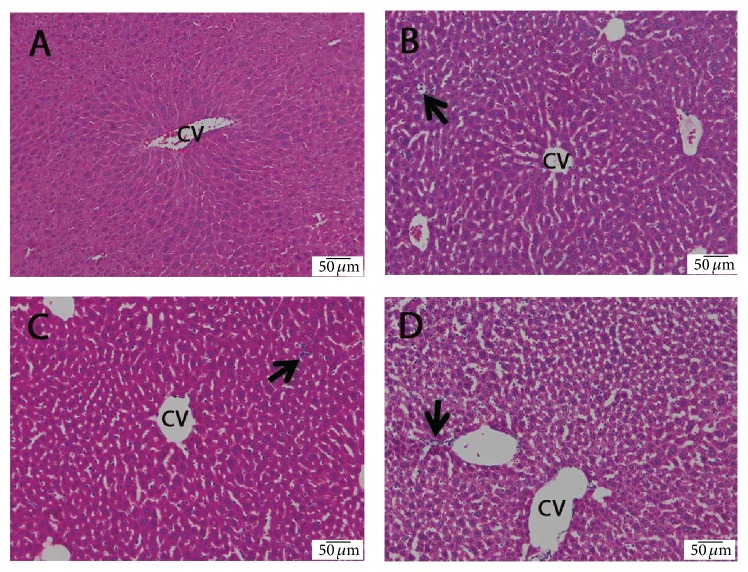
The effect of flucytosine and amphotericin B coadministration on histological changes in the livers of experimental mice: (A) control group; (B) 50 mg/kg flucytosine + 300 *μ*g/kg amphotericin B group; (C) 100 mg/kg flucytosine + 600 *μ*g/kg amphotericin B group; (D) 150 mg/kg flucytosine + 900 *μ*g/kg amphotericin B group. CV: centrilobular vein; arrow: inflammatory cell infiltration.

Liver sections from control mice showed normal liver architecture (Figure 1(A)). In the flucytosine-amphotericin B groups, sinusoidal dilatation, inflammatory cell infiltration, and vascular congestion were present (Figure 1(B–D)). The structural changes were dose-dependent.

### 3.2. Flucytosine and Amphotericin B Coadministration Upregulates Production of Hepatic Proinflammatory Cytokines

**Figure 2:**
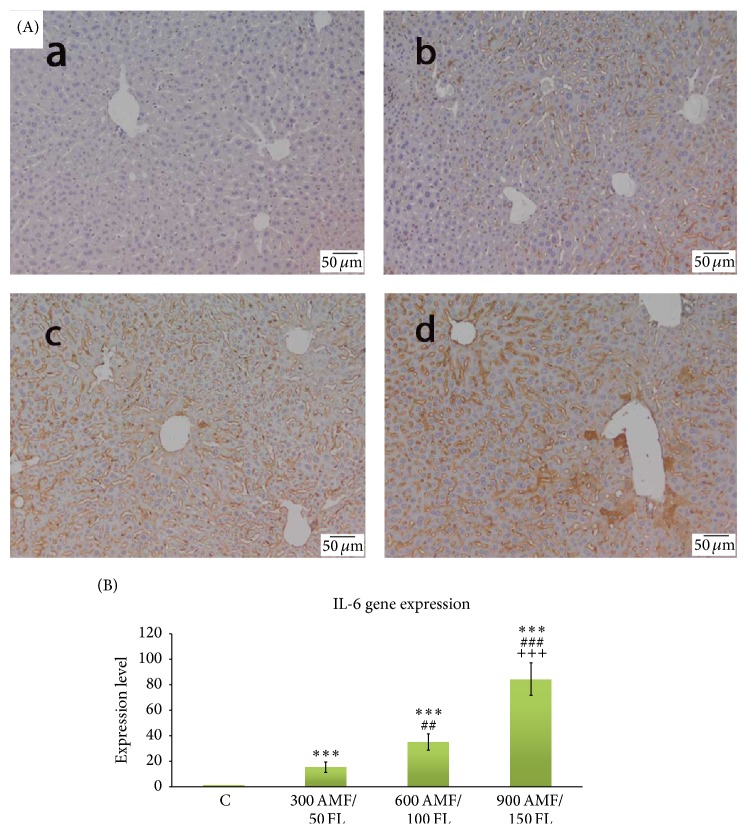
The effect of flucytosine and amphotericin B coadministration on the gene expression and specific hepatic distribution of IL-6. (A): (a) control group; (b) 50 mg/kg flucytosine + 300 *μ*g/kg amphotericin B group; (c) 100 mg/kg flucytosine + 600 *μ*g/kg amphotericin B group; (d) 150 mg/kg flucytosine + 900 *μ*g/kg amphotericin B group; (B) real-time PCR was used to investigate mRNA levels of IL-6 target genes. Significance levels are marked as follows: ^*∗∗∗*^ *P* < 0.001 compared with control, ##<0.01 and ^###^ *P* < 0.001 compared with 300 AMF/50 FL group, and ^+++^ *P* < 0.001 compared with 600 AMF/50 FL group.

The effects of flucytosine and amphotericin B coadministration on the IL-6 expression are shown in Figure 2. The livers of control mice did not show substantial IL-6 immunopositivity (Figure 2 A(a)). Flucytosine-amphotericin B cotreatment increased the number of cells labeled with IL-6 antibody in dose-dependent manner (Figure 2 A(b–d)). The results obtained by immunohistochemistry were confirmed by the results of IL-6 gene expression analysis, where IL-6 levels significantly increased with 15.16%, 35.08%, and 84.32% for antifungal cotreated groups as compared to the corresponding levels in the control group (Figure 2(B)).

**Figure 3:**
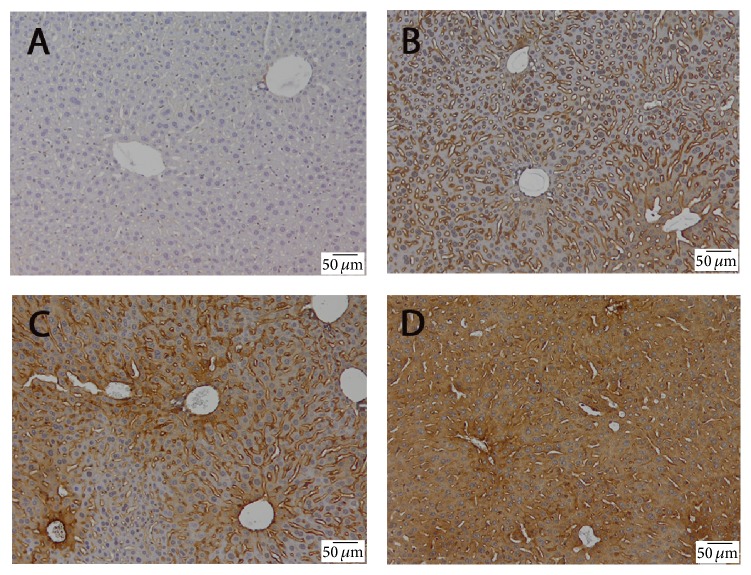
The effect of flucytosine and amphotericin B coadministration on the expression and specific hepatic distribution of TNF-*α*: (A) control group; (B) 50 mg/kg flucytosine + 300 *μ*g/kg amphotericin B group; (C) 100 mg/kg flucytosine + 600 *μ*g/kg amphotericin B group; (D) 150 mg/kg flucytosine + 900 *μ*g/kg amphotericin B group.

TNF-*α* expression was found in the treated groups (Figure 3). TNF-*α* immunoreactivity was observed mainly around centrilobular areas. No apparent TNF-*α* expression was detected in nonparenchymal cells. Treatment with antifungal drugs resulted in an increase of TNF-*α* expression in a dose-dependent manner, being the most prominent in mice treated with 150 mg/kg flucytosine + 900 *μ*g/kg amphotericin B (Figure 3(D)).

### 3.3. Effect of Flucytosine and Amphotericin B Coadministration on the Activation of NF-*κ*B

**Figure 4:**
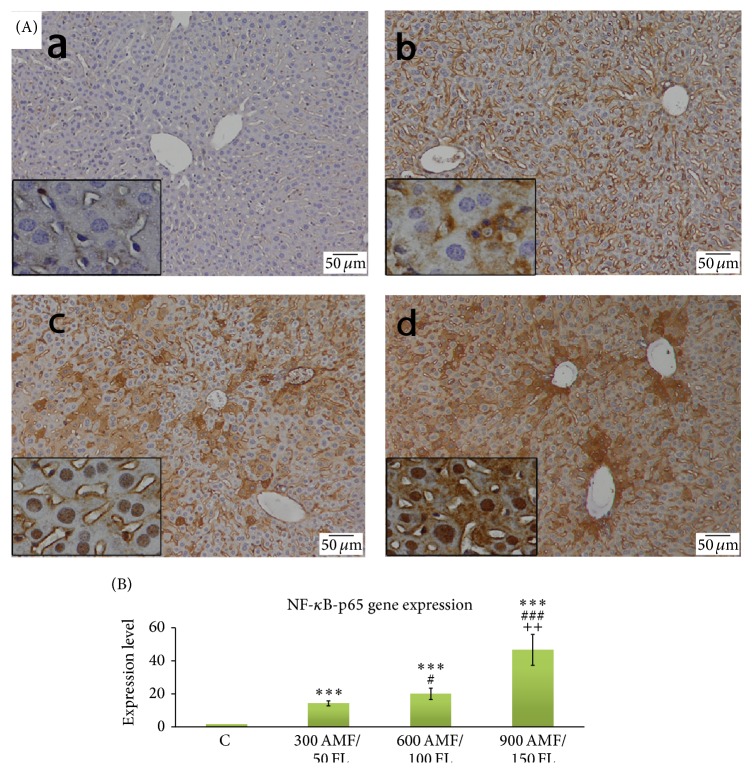
The effect of flucytosine and amphotericin B coadministration on the gene expression and specific hepatic distribution of NF-*κ*B p65 (A): (a) control group; (b) 50 mg/kg flucytosine + 300 *μ*g/kg amphotericin B group; (c) 100 mg/kg flucytosine + 600 *μ*g/kg amphotericin B group; (d) 150 mg/kg flucytosine + 900 *μ*g/kg amphotericin B group; (B) real-time PCR was used to investigate mRNA levels of IL-6 target genes. Significance levels are marked as follows: ^*∗∗∗*^ *P* < 0.001 compared with control, #<0.05 and ^###^ *P* < 0.001 compared with 300 AMF/50 FL group, and ^++^ *P* < 0.01 compared with 600 AMF/50 FL group.

The livers of control mice did not show substantial NF-*κ*B immunopositivity (Figure 4 A(a)). Liver sections from mice coadministered with flucytosine and amphotericin B have shown strong immunopositivity, remarkably in the periportal and perilobular areas (Figure 4 A(b–d)). The results were similar for mRNA levels of NF-*κ*B: they were significantly increased for 50/300, 100/600, and 150/600 of flucytosine-amphotericin B coadministered groups 13.88-, 19.85-, and 46.61-fold, respectively, compared to control group (Figure 4(B)).

### 3.4. Flucytosine and Amphotericin B Coadministration on the Activation of Kupffer Cells

Mouse Kupffer cells are commonly identified using the F4/80 marker [14].

**Figure 5:**
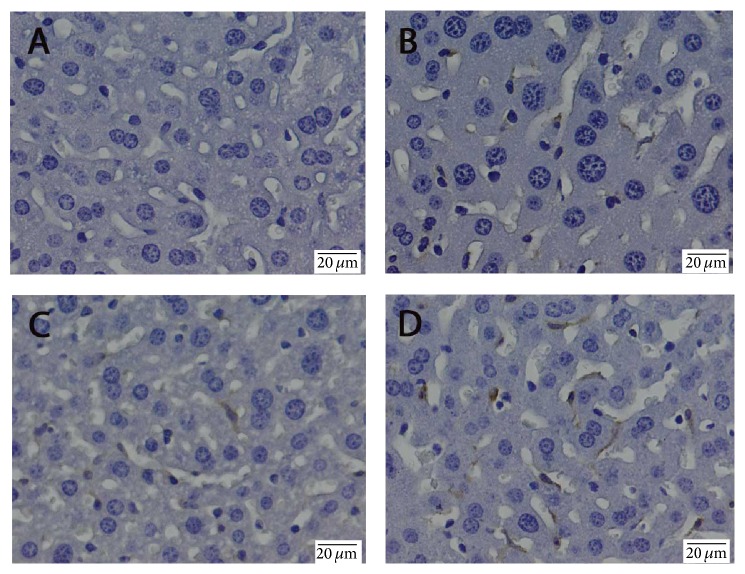
The effect of flucytosine and amphotericin B coadministration on the expression and specific hepatic distribution of F4/80: (A) control group; (B) 50 mg/kg flucytosine + 300 *μ*g/kg amphotericin B group; (C) 100 mg/kg flucytosine + 600 *μ*g/kg amphotericin B group; (D) 150 mg/kg flucytosine + 900 *μ*g/kg amphotericin B group.

Detection of Kupffer cells by immunohistochemistry after antifungal treatment showed an increase in number of F4/80^+^ cells in dose-dependent manner (Figure 5).

**Figure 6:**
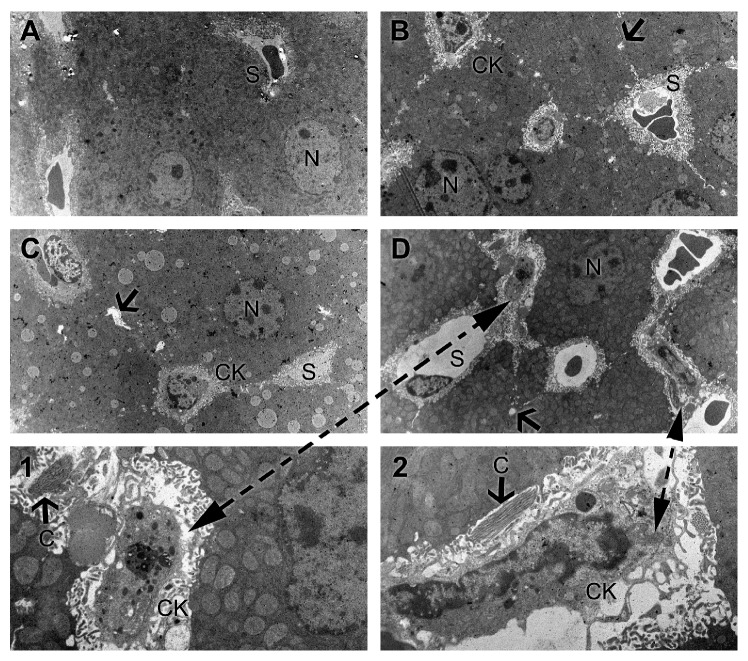
The effect of flucytosine and amphotericin B coadministration on ultrastructural changes in the livers of experimental mice: (A) control group; (B) 50 mg/kg flucytosine + 300 *μ*g/kg amphotericin B group; (C) 100 mg/kg flucytosine + 600 *μ*g/kg amphotericin B group; (D) 150 mg/kg flucytosine + 900 *μ*g/kg amphotericin B group. (1, 2) Detail of activated Kuppfer cells and perisinusoidal fibrosis. N: nucleus, CK: Kuppfer cells, S: sinusoids, C: collagen, and arrow: dilated bile canaliculi.

Electron microscopy investigations of experimental livers evidenced a normal ultrastructural aspect in the control group (Figure 6(A)). Antifungal coadministration causes dilatation of sinusoids and bile canaliculi, which join each other to form enlarged structures, devoid of microvilli, compared to control. These changes are dose-dependent. Kupffer cells became activated at highest dose of combined antifungal therapy, noticed by intense phagocytic activity (Figure 6 D(1-2)). Also, perisinusoidal fibrosis was present (Figure 6(D2)).

## 4. Discussion

Higher rates of mycoses are reported in hospitals, as a result of the increasing percent of immunocompromised patients at risk for invasive fungal infections, which in many cases are associated with high mortality rates and health care costs [15]. Furthermore, the common practice is to switch to a different class of antifungal agents or to combine therapies for the patients who fail the primary therapy or who experience intolerable adverse reactions [8].

Combined therapy of flucytosine and amphotericin B is the recommended first-line treatment for many invasive fungal infections, as disseminated cryptococcosis in both immunocompetent and immunosuppressed patients [7]. Because the side-effects of both monotherapies were established, in this study we aimed to evaluate the hepatotoxicity induced by combined therapy at increasing doses.

Acute hepatotoxicity induces inflammation and hepatocyte apoptosis or necrosis. In many cases, cell death is not owed to the damaging agent itself, but to the inflammation results. Histological analysis revealed an increase of parenchymal and portal inflammation in mice, in dose-dependent manner. The inflammatory infiltrate seen in histological liver sections is a naturally occurring reaction of the system to damaged cells [16].

Hepatic inflammatory response is mediated by the proinflammatory cytokines, especially tumor necrosis factor-*α* (TNF-*α*), which can further modulate the effects of other cytokines, as IL-1, IL-2, IL-4, IL-6, IL-10, IL-12, IL-18, and IL-23 [17]. Although almost detectable in the normal liver, production of TNF-*α* is one of the first events in many types of liver injury. As expected, in our mouse model, amphotericin B and flucytosine coadministration stimulated activation of an inflammatory pathway, demonstrated by a significant increase of TNF-*α* immunoreactivity. This was dose-dependently increased by 50/300, 100/600, and 150/600 of flucytosine-amphotericin B cotreatment, suggesting that combined therapy exerts a synergistic inflammatory activation in a dose-dependent manner. Subsequently, activation of TNF receptors leads to induction of death signals, TNF-*α* being primarily involved in juxtacrine signaling of cytotoxicity. It has been shown recently that critical drug-induced toxicity pathways act in synergy with the proinflammatory TNF-*α* [18]. TNF-*α* is a powerful promoter of the inflammatory response, directly or through stimulation of proinflammatory mediators, including IL-6 secreted by Kupffer cells. IL-6 is the predominant regulator of the hepatic acute-phase response and modulates liver fibrosis through degrading extracellular matrix proteins by inhibition of proteases or by binding to other cytokines [19, 20]. Our study showed that IL-6 is stimulatory as suggested by higher levels in hepatic tissue and upregulation of target genes in mice with amphotericin B and flucytosine-induced liver inflammation. Similar trends were observed by Cho et al. [21] and Xiao et al. [22] for other xenobiotics.

NF-*κ*B is a key transcription factor that plays a crucial role in the cellular response to signal transduction by TNF-*α* and IL-6 and is thereby involved in several signal transduction pathways of inflammatory response by controlling the expression of genes encoding cytokines [23]. NF-*κ*B is also known to activate the transcription of TNF-*α*-inducible genes, including IL-1 [24]. Thus, TNF-*α* and IL-1*β* promote NF-*κ*B activation, both leading to enhanced production of supplementary proinflammatory cytokines. Following cellular stimulation, the I-kappaBs inhibitors, which in normal conditions sequester p50/p65 dimers of NF-*κ*B in the cytoplasm, are rapidly degraded, activating NF-*κ*B. The active form p65 of NF-*κ*B rapidly translocates into the nucleus, binding to consensus sequences in the promoter/enhancer region of various genes, promoting their transcription, and regulating various inflammatory responses. Prolonged activation of NF-*κ*B leads to perpetuated inflammatory response, which induced liver injury. This cycle can affect the structure and ultrastructure of the hepatocytes and impairs their function accordingly. In our study, NF-*κ*B p65 was activated after antifungal treatment, demonstrated by dose-dependent increased immunohistochemical expression and upregulation of target genes (Figure 4).

Kupffer cells are resident macrophages of the liver and play an important role in the pathogenesis of liver injury mediated by chemical substances, toxins, and pharmacological agents. Activation of Kupffer cells by toxic agents results in the release of inflammatory mediators, growth factors, and reactive oxygen species and further modulates acute hepatocyte injury [25].

In response to proinflammatory signals of antifungal administration, Kupffer cells become activated, revealed by increased number of F4/80^+^ cells (Figure 5).

In addition, ultrastructural analysis strengthens immunohistochemical observations, showing that the Kupffer cells from antifungal treated livers were enlarged with increased secretory vesicles and phagocytic activity (Figure 6). Direct or indirect activation of Kupffer cells by toxic agents results in the release of an array of inflammatory mediators including cytokines, growth factors, and reactive oxygen species [26]. This activation appears to modulate acute hepatocyte injury [25], noticed by us through increased production of TNF-*α* and IL-6 under combined flucytosine-amphotericin B treatment, in a dose-dependent manner (Figures 2 and 3).

## 5. Conclusions

Our results suggest that antifungal combined therapy exerts a synergistic inflammatory activation in a dose-dependent manner, through NF-*κ*B pathway, which promotes an inflammatory cascade during inflammation.

The use of amphotericin B and flucytosine in combined therapy needs to be dose limiting due to the associated risk of liver injury, especially for those patients with hepatic dysfunction, requiring careful monitoring along antifungal therapy.

## Figures and Tables

**Table 1:** Primer sequences used in the study.

Code	Sense	Antisense
GAPDH	CGACTTCAACAGCAACTCCCACTCTTCC	TGGGTGGTCCAGGGTTTCTTACTCCTT
IL-6	AAAGAGTTGTGCAATGGCAATTCT	AAGTGCATCATCGTTGTTCATACA
NF-*κ*B p65	CTTGGCAACAGCACAGACC	GAGAAGTCCATGTCCGCAAT
